# Application of whole genome shotgun sequencing for detection and characterization of genetically modified organisms and derived products

**DOI:** 10.1007/s00216-016-9549-1

**Published:** 2016-04-21

**Authors:** Arne Holst-Jensen, Bjørn Spilsberg, Alfred J. Arulandhu, Esther Kok, Jianxin Shi, Jana Zel

**Affiliations:** Norwegian Veterinary Institute, Ullevaalsveien 68, P.O. Box 750, Sentrum, 0106 Oslo Norway; RIKILT, Wageningen UR, P.O. Box 230, 6700 AE Wageningen, The Netherlands; Joint International Research Laboratory of Metabolic & Developmental Sciences, Shanghai Jiao Tong University–University of Adelaide Joint Centre for Agriculture and Health, School of Life Sciences and Biotechnology, Shanghai Jiao Tong University, Shanghai, 200240 People’s Republic of China; National Institute of Biology, Večna pot 111, 1000 Ljubljana, Slovenia

**Keywords:** Cisgene, Intragene, Traceability, Transcriptome sequencing, Transgene, Unknown GMO

## Abstract

The emergence of high-throughput, massive or next-generation sequencing technologies has created a completely new foundation for molecular analyses. Various selective enrichment processes are commonly applied to facilitate detection of predefined (known) targets. Such approaches, however, inevitably introduce a bias and are prone to miss unknown targets. Here we review the application of high-throughput sequencing technologies and the preparation of fit-for-purpose whole genome shotgun sequencing libraries for the detection and characterization of genetically modified and derived products. The potential impact of these new sequencing technologies for the characterization, breeding selection, risk assessment, and traceability of genetically modified organisms and genetically modified products is yet to be fully acknowledged. The published literature is reviewed, and the prospects for future developments and use of the new sequencing technologies for these purposes are discussed.

## Introduction

First-generation sequencing technology [1] revolutionized genetics and brought about the sequencing of the first large eukaryote genomes (human [2] and mouse [3]), and later the first crop plant genomes (rice [4] and soybean [5]). These genomes laid the foundation for detailed genetic studies of these organisms. Next-generation sequencing (NGS) with a concurrent rapid drop in cost per base pair and increase in throughput accelerated this development. This evolution continues, and the NGS technologies are gradually challenged by so-called third-generation sequencing technologies [6]. In this review, NGS and third-generation sequencing technologies will jointly be referred to as “high-throughput sequencing (HTS) technologies.” HTS has recently paved the way for whole genome sequencing (WGS) of the most important food crops, including maize [7] and the hexaploid wheat [8], and the most important farm animals, such as sheep [9], pigs [10], and cattle [11]. The information found in sequenced genomes is used, for example, by breeders to identify markers for desired traits [12, 13] and to better understand the effects of potential modulations of synthetic pathways, including genetic modifications.

### Functional effects and reasons to characterize genetic modifications by sequencing

Genetic modifications are by definition modifications of nucleic acids, and are intended to yield altered functional characteristics of the modified organism. Whether or not the modifications have to be inheritable depends on the jurisdiction. In the European Union (EU), for example, the definition includes inheritability [14], whereas in Norway, for example, the definition does not include inheritability [15]. Some genetic modifications are desired, others are unintended and potentially harmful or unexpected. For developers of genetically modified (GM) organisms (GMOs) it is desirable to have detailed information about the characteristics, both genetically and phenotypically, of the GMOs they develop and the corresponding non-GM parental lines. This facilitates comparison and selection among the GM lines for further breeding and commercialization. To safeguard health and the environment, and to provide consumers with a freedom of choice and inform society, many countries regulate the commercialization of GM products and the release of GMOs into the environment. Regulations usually include requirements for substantial risk assessment before field trials, release into the environment, and use as food and feed. Authorization (in most countries, including members of the EU) or deregulation (in the USA) is successively required for the use of GMOs and derived products in foods and feeds. Detailed descriptions of the genetic modifications are fundamental raw data to be included as one part of the total documentation required for these risk assessments [16].

### Target motifs for detection of GMOs

**Fig. 1:**
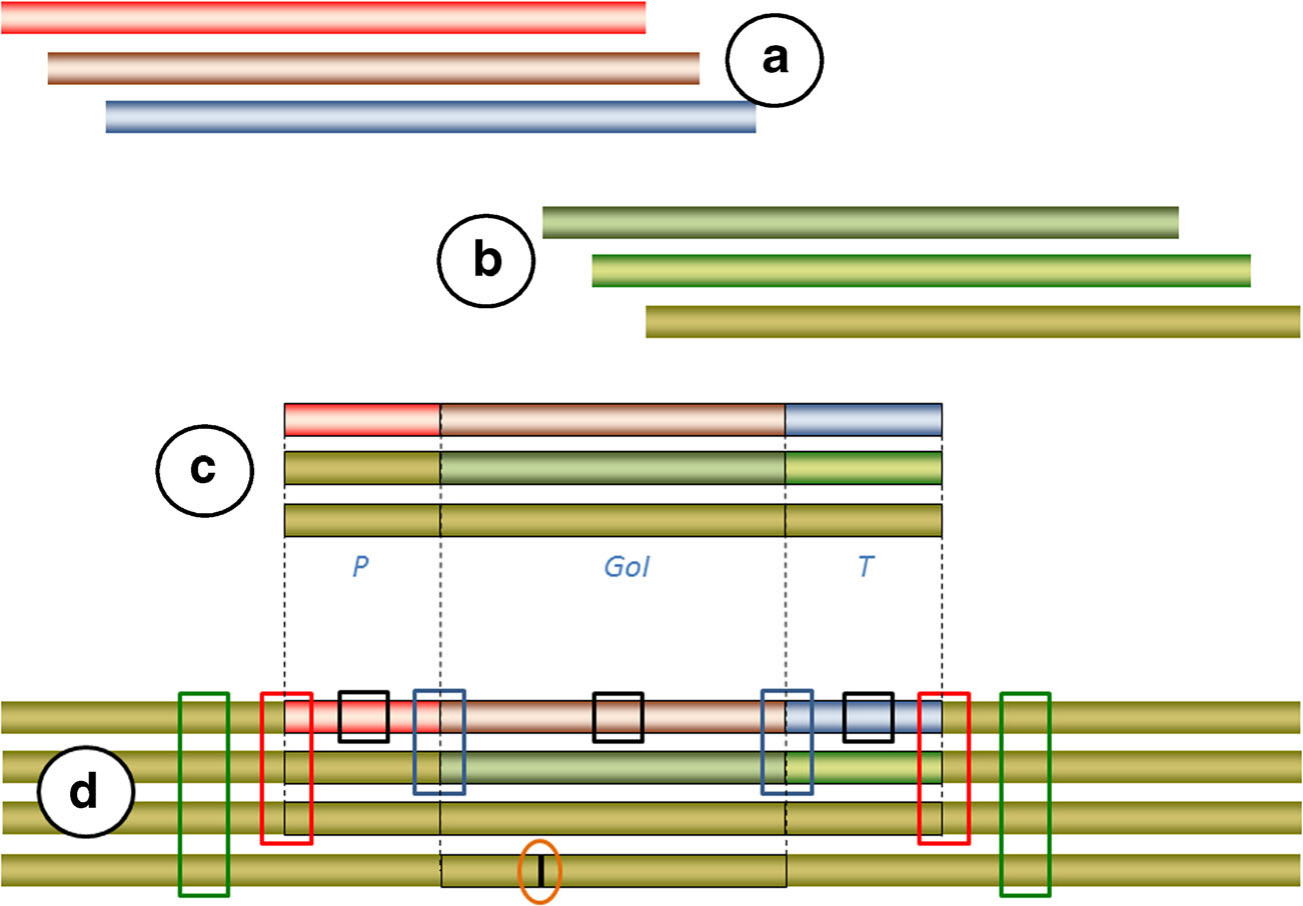
The relationships between genetic modifications and sequence motifs suitable for detection and identification of genetically modifies organisms (GMOs). *A:* Chromosomes of taxa distantly related to the genetically modified taxon (e.g., *red* for virus, *brown* for fungus, *blue* for bacterium). These are sources of transgenic sequence motifs. *B*: Chromosomes of the modified species or closely related, sexually compatible species (taxa; various *tones of green*). These are sources of intragenic and cisgenic sequence motifs. *C*: Genetic constructs (functional cassettes) as intended for insertion into the recipient organism, comprising (each element is framed) the promoter (*P*), gene of interest (trait gene; *GoI*), and terminator (*T*); *top* transgenic construct, *middle* intragenic construct (i.e., with elements combined into a nonnaturally occurring configuration), *bottom* cisgenic cassette (i.e., with original naturally occurring configuration). *D*: Genetic modification types as they appear in the recipient chromosomal locus: *top* transgenes, *upper middle* intragenes, *lower middle* cisgenes, bottom single nucleotide modification (*vertical black bar*). Four types of sequence motifs are commonly targeted for detection and identification of GMOs: screening elements (*open black boxe*s; a single element of a construct), construct-specific junctions (*open blue boxes*; motif is a chimera of two different elements of the construct), event-specific junctions (*open red boxes*; motif is a chimera of the insert and the native insertion locus), and taxon-specific motifs (*open green boxes*; typically a single copy housekeeping gene serving as a reference gene for identification and quantification of the modified taxon). The number of alternative targets that can be used to detect GMOs decreases as one move from transgenes (event-, construct-, and element-specific motifs) via intragenes (event- and construct-specific motifs) to cisgenes (only event-specific motifs). This has a great impact on the approaches taken/required for GMO detection, and challenges the current paradigm of screening before identification and quantification. Single nucleotide modifications and naturally occurring single nucleotide polymorphisms are indistinguishable from each other (*orange oval*). Rearrangements of the inserted genetic construct and native genome are not uncommon (not shown)

The DNA sequences of the genetic modification are the targets for detection of GMOs and derived products. The first generation of GMOs were modified by insertion of DNA constructs composed of genetic elements (promoters, genes, and terminators) from species other than the recipient taxon (transgenes). The insertion locus, the number of copies of inserted DNA, and the structure of the inserted DNA were rather unpredictable, and rearrangements of the recipient genome and insert construct(s) were common. With time, however, the number of copies, the structure of inserts, and the insertion loci have become much more predictable and controllable. The functionality of a genetic construct is affected by the choice of promoter, gene, and terminator. It is often desirable that the novel trait is expressed only under particular conditions or in a particular tissue of the modified organism. This requires the use of specific promoters from the modified taxon. Furthermore, codon usage and posttranscriptional and posttranslational modification are often much better when the coding gene is derived from a closely related, sexually compatible species (taxon; intragene) or the modified species itself (cisgene). However, a transgene can also be modified to fit the codon preferences of the host. The distinction between transgene, intragene, and cisgene is explained in Fig. 1. Although the insertion of a functionally coding construct is still the rule among developers of GMOs, new emerging gene technologies now make it possible to modify only a single nucleotide (single nucleotide modification, SNM), for example, by application of CRISPR–Cas9 systems; see, for example, [17–21]. This represents a paradigm shift for the detectability of GMOs (Fig. 1).

### Unknown structural changes and insertions in genomes

Natural cell division, recombination, and directed breeding processes result in unintended structural variants, including single nucleotide substitutions [single nucleotide polymorphisms (SNPs) corresponding to SNMs], deletions, insertions, duplications, and rearrangements in a genome. Viral infections, transposons, and rare events of horizontal gene transfer also result in the presence of unknown insertions and structural changes in genomes. Examples in the literature demonstrate the relevance of molecular characterization as a basis for comparative risk assessment [17, 22–25]. They also, as exemplified and discussed later, provide tools suitable for characterization of unknown and unintended genetic modifications in transgenes, intragenes, and cisgenes, as well as detection of SNPs/SNMs (see, e.g., [26]).

### Classification of GMOs on the basis of available sequence information

Holst-Jensen et al. [27] classified GMOs into four insert sequence knowledge (ISK) classes on the basis of the sequence information available a priori. For the purpose of selecting the best approach for characterization and detection of GMOs, it is very helpful to understand and apply this classification (Table 1).

**Table 1:** Insert sequence knowledge (*ISK*) classes

Class	Short description	Examples
ISK-1	A GMO where the complete insert and event-specific junction sequences are known	GMOs authorized in the EU (EU register of authorized GMOs; http://ec.europa.eu/food/dyna/gm_register/index_en.cfm)
ISK-2	A GMO whose genetic modification is not fully sequence characterized but the sequence of the construct intended for insertion is known	Sister events to GMOs authorized in the EU, such as the maize event DAS-59132-8 (unauthorized in the EU and sister event to the EU-authorized maize event DAS-59122-7)
ISK-3	A GMO whose genetic modification is far from fully sequence characterized but the sequence of at least one element of the inserted construct is known	GMOs transformed with modified versions of broadly applied vectors such as the pCAMBIA vectors (for plants) and pcDNA vectors (for mammals), where at least one vector-derived element is present in the GMO
ISK-4	A GMO whose genetic modification contains no element present in the genetic modification of a GMO belonging to one of the other ISK classes	A GMO with a completely novel inserted construct and no vector-derived elements/motifs. Some information (e.g., on the donor species or phenotypic function of the insert) may still be available.

Modified from [27]

*EU* European Union, *GMO* genetically modified organism

### Available WGS technologies

Several distinct HTS platforms have emerged since the turn of the millennium. Companies with unique platforms compete to offer higher throughput and more reliable sequence data that can be obtained faster and more cheaply. The most important differences between the platforms are associated with the input nucleic acid requirements and the output read length, total number of sequenced bases, error rate, runtime and cost per sequenced million bases. This means that the different platforms also have different advantages and disadvantages, and are fit for different purposes (Table 2). The sample preparation strategy to be applied is tightly connected to the purpose and thus to the platform selected for a given case.

**Table 2:** Leading commercial sequencing platforms

Platform	Output read length	Output no. of reads	Runtime	Type of reads	Comments
Illumina HiSeq	100–150 bp	≤350 million/lane (8 lanes/run)	1–6 days	Paired end	Currently the dominating platform on the market. Insert size^a^ 300–600 bp. Lowest cost per sequenced base pair. Sequencing by synthesis
	100–150 bp	≤350 million/lane (8 lanes/run)	1–6 days	Mate pair	Insert size^a^ up to several thousand base pairs. Significantly higher costs for library preparation compared with paired-end sequencing
Illumina MiSeq	≤300 bp	≤25 million/run	Hours to 3 days^b^	Paired end/mate pair	Insert size^a^ and principle as for HiSeq
Oxford Nanopore Technologies MinION^c^	≤200 kbp	≤2.5 million at 10 kb and standard speed	Minutes to 48 h (sequencing in real time)	Single molecule	Highly flexible read length. Low cost per run. High error rate requires high coverage to obtain consensus sequence. Nanopore sequencing
Pacific Biosciences PacBio	>10 kbp	500 Mbp to 1Gbp	0.5–6 h/SMRT cell, 1–16 cells/run	Single molecule	Read length highly dependent on input DNA. High cost per sequenced base pair. Single-molecule real-time sequencing with zero-mode waveguide
Roche 454	≤800 bp	≤100,000	18 h	Single end	Withdrawn from the commercial market in 2015/2016. Widely used in studies requiring longer reads. Pyrosequencing.
Thermo Fisher Ion Torrent	≤400 bp	≤80 million/run	A few hours	Single end	Low throughput, low cost per run. Ion semiconductor sequencing

^a^Insert size is the length of two reads plus the distance in base pairs between them.

^b^Runtime is largely dependent on the number of cycles (length of reads).

^c^Read length, runtime, and base calling accuracy are independent according to the manufacturer.

WGS generates very large datasets. The main challenge is not the generation of sequence data but the subsequent data analysis. Targeting the analyses to detect only a predefined set of sequence motifs or SNPs/SNMs is relatively simple and fast and therefore potentially cost-effective in comparison with a broader nontargeted approach. The drawbacks are primarily the bias and inability to detect unknown targets (inserted, recombined, and rearranged sequences). Unbiased analyses to detect all structural variants, novel insertions, and broad ranges of SNPs/SNMs in relevant coding genes, on the other hand, require substantially more resources, limiting the applicability of such approaches. Presequencing enrichment approaches and their application in combination with new sequencing technologies are reviewed elsewhere (Arulandhu et al., this volume). The present review aims to (1) highlight the needs for detection, identification, and characterization of genetic modifications, GMOs, and derived materials, (2) review the available literature related to the use of WGS technologies and tools for unbiased, nontargeted purposes, (3) identify challenges and gaps in the availability of applicable solutions in relation to the needs identified, and (4) discuss prospects for further developments and improvements that can mitigate challenges and perhaps close the identified gaps.

## Comparative approaches to identify genetic modifications

Breeding, including the development of GMOs, results in the development of new, putatively stable lines, genetically distinct from the parental lines. Theoretically, the distinctive genetic characteristics can therefore be identified by comparison of the completely sequenced genome of any GMO with its isogenic non-GM counterpart (parental line) or reference genome. Tissue culturing of plants often results in somaclonal variation (i.e., genetic or phenotypic variation) not resulting from genetic modification or sexual recombination [28]. Genetic somaclonal variation can make comparisons of GM and isogenic counterparts more difficult [25]. From a legal point of view, natural genetic variation, including somaclonal variation, should not be confused with gene-technology-derived genetic variation (i.e., the basis for classification of a plant or animal as a GMO [14, 29, 30]). The ability to assess this difference is therefore required. With the availability of fully sequenced genomes (approximately 100 higher plants, more than 40 mammals, more than 50 birds, etc.), it is possible to compare the sequence of a sample with the reference sequence(s) in databases. Such a resequencing approach is particularly useful if the databases contain large numbers of relevant accessions. An example is that fully sequenced genomes of many rice cultivars representing the majority of the global genetic variation of rice are available for comparison with a sequenced (putative) GM rice sample [31].

**Fig. 2:**
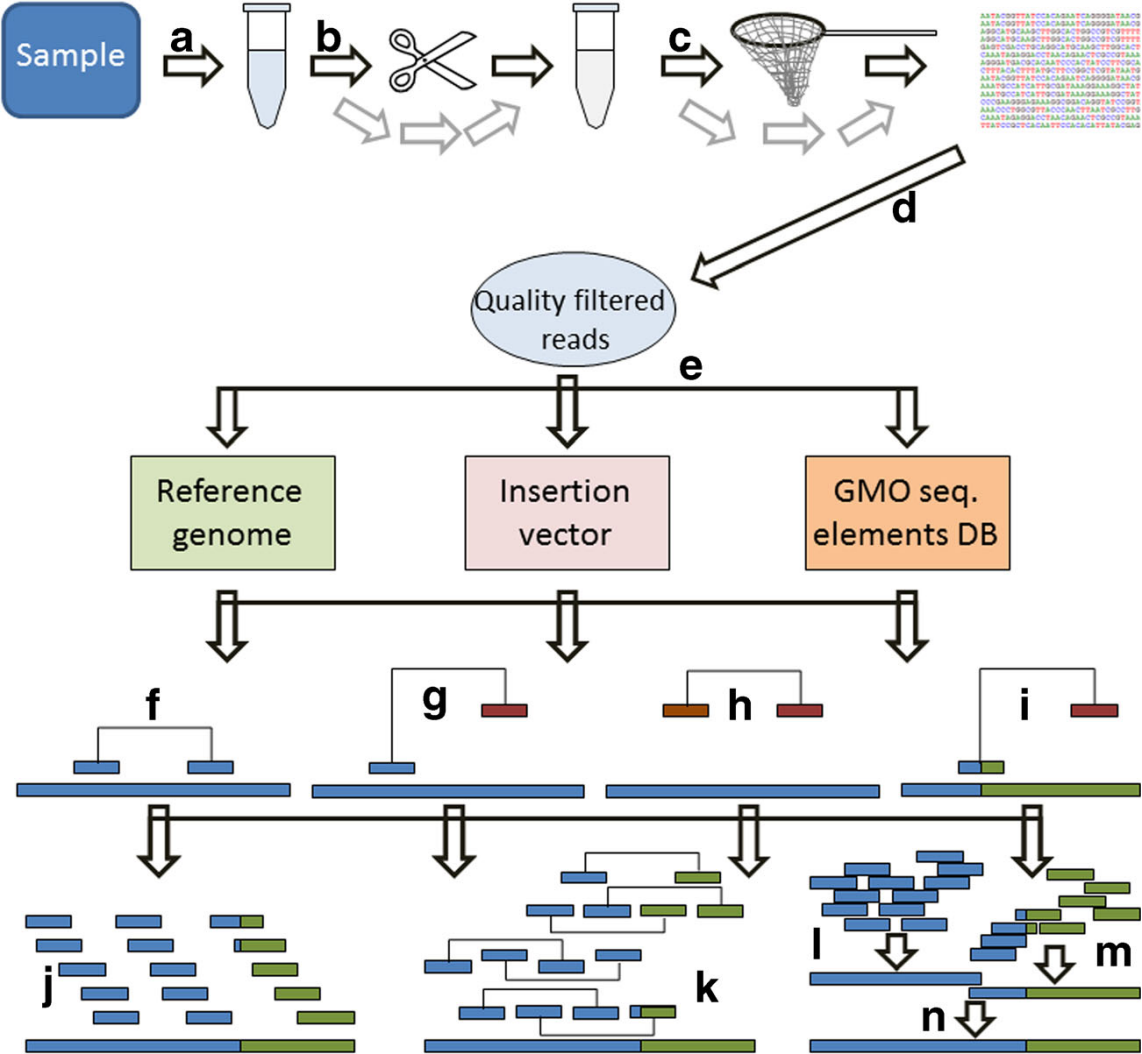
Approaches to detect, characterize, and identify GMOs by application of whole genome shotgun sequencing. First the DNA is extracted from the sample and purified (*A*). Longer fragments are optionally split into shorter fragments to obtain a sequencing library with fragments of a desired size range; for example, approximately 500 bp for paired-end sequencing or 2–10 kbp for mate-pair sequencing (*B*). Capture enrichment from the sequencing library can optionally be performed before sequencing. The order of fragmentation and enrichment can be switched (*C*).Raw sequencing reads are quality filtered before bioinformatics analysis (*D*). Three types of available sequence databases can be available for GMO analyses (*E*): the full sequence of the taxon (reference genome), the full sequence of the insertion vector used to create the GMO, and a collection (more or less complete) of sequence elements/motifs associated with various GMOs (GMO sequence elements database). Mapping of the reads to these databases can result in identification of *F* perfect matches (i.e., concordant mapping), *G* reads with matching and orphan mates (i.e., discordant mapping), *H* nonmapping (i.e., unmapped reads), and *I* chimeric reads mapping partially to one database sequence and partially to another database sequence (i.e., split reads). The illustrated orphan reads in *G* and *I* sometimes map to other sequences in the same or other databases, and in such cases provide useful information for further mapping. Notably, some sequencing technologies produce single reads, not paired/mated reads. In these cases only *F*, *H*, and *I* can be observed. Mapping can be done against only one of the references or against two or all databases. Depending on the order and results of the mapping, the outcome is usually a subset of sequence reads that can be used to infer the sequence of the genetically modified insert and its insertion locus. Perfectly mapped reads confirm the presence of a particular sequence motif (*J*). Paired/mated reads can facilitate the assembly of reads into contigs (*K*). Single reads can be assembled to shorter contigs (*L* and *M*) that can successively be assembled into longer contigs (*N*). *DB* database, *seq.* sequence

Transgene insertion, which is presently the commonest type of genetic modification among commercialized GMOs (mainly plants), involves the insertion of “foreign” genetic material. This insertion is characterized by two features (Fig. 1): (1) the presence of “foreign” genetic material which is absent in the native genome and (2) the creation of unique chimeric sequence motifs at each end of the insert. This creates the possibility of at least two approaches to detection of genetic modifications by bioinformatics sequence analysis (Fig. 2). First, by subtraction of all sequence motifs that match the native (non-GM) genome, only a subset of sequence reads are retained for further analysis. These reads can be de novo assembled into contigs but this is optional before further analyses. The retained reads and/or assembled contigs can be compared with sequence databases to search for identical or similar sequence motifs. Searching for open reading frames (ORFs) in contigs can potentially reveal protein-coding genes. Such analyses can provide information about the novel (transgenic) trait(s), which is important, among other reasons, for risk assessment. Suitable sequence databases include databases dedicated to sequence motifs known to be present in existing GMOs—for example, the GMO Detection Method Database (GMDD) [32]—or more general databases (e.g., GenBank [33]). The EUginius database (http:// www.euginius.eu) can successively be used when one or more elements are identified to list candidate GMOs containing the element(s). Secondly, sequence motifs that only partially match the native genome represent putative breakpoints (i.e., positions where it is likely that insertion or rearrangement of the genome has occurred). Further analysis of these putative breakpoints by comparison with sequences in the databases may identify the specific chimeric motifs associated with the insertion of a transgene (Fig. 1), and each such chimeric sequence motif, in turn, is a unique signature of a specific genetic modification event. The same approach can be taken to identify the presence of integrated viral or other horizontally transferred genetic elements [34–36]. Many transgenic plants contain in their novel inserts one or more transgenic elements that are found also in other transgenic plants [37]. In animals, functional constraints on transgenic elements are more pronounced, and therefore fewer elements are used repeatedly in different GM animals than in GM plants. The common presence of the same transgenic element(s) in GM plants is the basis for the application of element screening, often referred to as the matrix approach in polymerase chain reaction (PCR)-based GMO testing [27, 37–40]. An alternative to subtraction of the native genome sequences is therefore to start by looking for sequence motifs matching GMO-associated motifs (e.g., the GMDD accessions), in what would be a sequencing-based matrix approach. This can be relatively fast and may be cost-effective, but like other targeted approaches is biased and would inevitably fail to detect GMOs belonging to ISK-4. Yet, combined with bioinformatics approaches to sequence assembly and/or gene walking approaches, it can be feasible to detect and characterize GMOs for which sequence data are missing/incomplete. That, in turn, would provide data useful for risk assessment [16] and development/application of specific PCR detection methods [27]. PCR-based detection is faster, more cost-effective, and more applicable for routine and on-site application than HTS if the number of targets is low and they are well characterized and their sequences are invariant. With growing numbers and less well characterized and heterogeneous targets, HTS becomes more attractive. Many genetic modifications introduced into plants are not found in the GMDD. For animals it is the rule that the matrix approach is unlikely to lead to detection and identification of a GMO. However, if additional information about the genetic modification is available before the analysis is set up, then a similar or semitargeted analysis can be performed. For example, if it is known that the genetic modification affects a particular metabolic pathway, then an option could be to direct and perform a semitargeted test at the genes involved/associated with the pathway.

### Target enrichment approaches

The specific sequence representing a genetic modification is only a minute fraction of the whole genome; for example, 6000 bp in a 3-Gbp genome (0.0002 %). It is therefore attractive to consider approaches to enrich the fraction of obtained sequence reads derived from the genetic modification, before analysis. One approach is to perform selective amplification; for example, by use of PCR before sequencing (reviewed by Arulandhu et al., this volume). Another is to perform hybridization capture by use of probes (e.g., on a microarray or other solid support). A third is to analyze the transcriptome, which represents only the transcriptionally active fraction of the genome in a particular organ or tissue. All these alternatives are biased and may result in incomplete detection/identification or even false negatives.

### De novo assembly and characterization of complete genomes with genetic modifications

De novo assembly of smaller genomes such as bacterial and fungal genomes is feasible in many cases. More than 1000 completely sequenced genomes of microorganisms have been published. The genomes of higher eukaryotes are much larger and more complex and therefore require substantially more effort to sequence and assemble them. De novo assembly of plant and animal genomes is therefore usually performed on one or a few selected (non-GM) reference lines/strains. Typically, efforts to obtain complete genome assemblies are organized in connection with international or regional collaborative programs for crop/livestock breeding/improvements [10, 41]. As a consequence, de novo assembly of a complete GM genome is only exceptionally required for ISK-4 scenarios, and various resequencing-based methods are attractive alternatives, as discussed later. However, de novo assembly of a moderately sized plant genome to a first draft quality is now feasible on a small computer cluster or even on a high-end, stand-alone machine.

The first transgenic plant genome to be fully sequenced and de novo assembled was the virus-resistant SunUp papaya genome [42]. Few details on the specific technology applied for the sequencing were provided, most likely because of interlaboratory variation in Sanger sequencing protocols. In that study, 2.8 million shotgun sequencing reads were obtained from an inbred papaya line [end sequencing of plasmid and bacterial artificial chromosome (BAC)-cloned fragments] and the main purpose was to characterize the papaya genome. A low average sequencing depth (approximately three times coverage) did not yield very detailed information on the transgene inserts and insertion loci. However, the evidence complemented by Southern blot data led to the conclusion that three inserts were present, and identified five of the six insert junctions as nuclear copies of papaya chloroplast DNA fragments. The supporting data for the study included transcript library data.

In September 2014, the presence of a viable GM microorganism (*Bacillus subtilis* overproducing riboflavin) in a food/feed additive was notified via the European Rapid Alert System for Food and Feed [43] (notification 2014-1249). This GM microorganism was not authorized for release in the EU, and no sequence information regarding the genetic modification was available. The GM microorganism was isolated and subsequently shotgun, whole genome sequenced by means of an Illumina HiSeq 2500 system by a Belgian–French team. The nearly 11 million paired-end reads (corresponding to a 350-fold coverage of the *B. subtilis* genome) were de novo assembled with CLC Genomics Workbench and scaffolded with SSPACE [44] into 36 scaffolds [45]. In a follow-up study [46], the team applied further bioinformatics analysis, exploiting the phenotypic information available (overproduction of riboflavin). This allowed the team not only to identify the biosynthesis operon and gene most likely subjected to genetic modification but also to identify nonnatural junction motifs including plasmid vector sequences in three of the contigs. Finally, after PCR and Sanger-sequencing-based verification of the presence of the inferred motifs in the GM microorganism, they developed a construct-specific real-time PCR detection method that can be applied for control purposes.

### Transcriptome sequencing analysis

The purpose of genetic modification is usually to modify the phenotype, via altered gene expression or introduction of a novel trait gene(s). Gene silencing is sometimes the intended modification, and silenced genes do not yield transcripts. In such cases, theoretically, quantitative analysis may exceptionally allow detection of GM cisgenes. Novel traits and strongly upregulated or downregulated gene expression, on the other hand, can be detected via the transcription of DNA to messenger RNA, provided that tissue-specific or environmentally determined variation in expression levels can be excluded. The transcriptome is therefore potentially an attractive target for detection and possible identification of a GMO. Comparative transcriptome sequencing can be applied to GMOs of any ISK class, making this one of the few available alternatives for ISK-4 scenarios. Transcription is affected by many factors, most importantly the promoter and transcription factors [47–49]. It is of critical importance that a transcriptome sample is taken from the right tissue at the right moment, as transcriptome-based GMO detection can otherwise be more prone to false negatives (i.e., GMO transcript not detected despite the presence of a GMO) than genome-based GMO detection. Furthermore, the transcripts by default do not include the full-length genetic construction defining the genetic modification or the event-specific junction sequence motifs required for unequivocal event-specific identification of GMOs (see Fig. 1). The transcriptome data can, however, be combined with successive genome walking strategies (see Arulandhu et al., this volume) to completely characterize each genetic construct and its associated event-specific junction motifs.

Tengs et al. [50] used computational subtraction to detect a transgene insert in *Arabidopsis thaliana* in an early pioneering work from 2009*.* The transcriptome (complementary DNA, cDNA) was sequenced with use of a Roche 454FLX instrument. The resulting 79,900 reads were mapped to the *A. thaliana* transcriptome and genome sequences by means of the Basic Local Alignment Search Tool (BLAST) [51], resulting in the retention of only 159 reads, of which 146 (approximately 92 %) matched the sequence of the pBI121 transfer DNA (T-DNA) *35S:GUS* Ti-plasmid vector that had been used to transform the transgenic *A. thaliana* line. The approach is applicable to transgenes, and possibly to intragenes, but not to cisgenic traits (where the transcript is also found in the non-GM). Tengs et al. [50] further explored the possibility to use computational subtraction (the bioinformatics component of the approach applied to the *A. thaliana* line) on two published expressed sequence tag libraries. One of these expressed sequence tag libraries was derived from the previously mentioned transgenic papaya study by Ming et al. [42], whereas the other was derived from a study of a transgenic rice line [52]. In both cases the approach successfully retrieved the sequence of an inserted transgenic novel trait gene.

The GM rice event OSCR11 was developed as a proof-of-concept edible vaccine for pollen allergy. Kawakatsu et al. [25] combined transcriptome and WGS analyses to compare this transgenic rice event with its non-GM parental line a123, with the aim of not only comparing the GM and non-GM lines but also of comparing traditional breeding with mutagenesis and radiation in molecular breeding. For this purpose, approximately 250 million paired-end reads were generated with use of an Illumina HiSeq 2000 instrument from each of the two rice lines. Similar data for the rice cultivar Koshihikari, which is the parental cultivar of a123, were downloaded from the National Center for Biotechnology Information Sequence Read Archive (previously known as the “Short Read Archive”). These three datasets were mapped to the rice reference genome [4, 53] with use of Burrows–Wheeler Aligner (BWA) in CLC Genomics Workbench before structural variant calling. Only one OSCR11-specific high-confidence structural variant was detected (i.e. a deletion of 810 bp). This structural variant was located in a repetitive sequence on chromosome 2, but it was not possible to dissect whether the deletion originated from the *Agrobacterium* transfection or not. SNP analysis with uniquely mapping reads analysis revealed 60,000–90,000 SNPs per line compared with the rice reference sequence. Of these SNPs, 167 were concluded to be induced by *Agrobacterium* transformation, and 939 were concluded to be induced by chemical- and radiation-induced mutagenesis. Insertion/deletion analysis revealed 1000–9000 sites compared with the rice reference genome, and 28 and 3147 were concluded to be transformation and mutagenesis induced respectively. From that data taken together, Kawakatsu et al. concluded that traditional mutagenesis induces more unintended mutations than molecular breeding, in agreement with an earlier array-based rice transcriptome study [22]. Strand-specific messenger RNA sequencing was performed with an Ion Torrent PGM instrument on an Ion 318 Chip and the sequence was analyzed for differentially expressed genes with the R package DESeq [54]. Twenty-eight differentially expressed genes were found, of which two were directly related to integrated T-DNA and another 12 were associated with endoplasmic reticulum stress and therefore believed to be indirectly linked to the transformation. By comparison with WGS data (discussed later), no transformation-induced SNPs or insertions/deletions were detected within 2 kbp upstream of any differentially expressed genes.

### Resequencing approaches

With access to a fully sequenced reference genome of the species in question, it is possible to compare the sequence reads obtained from WGS of the GMO or GM product with the reference genome, and then focus on the differences. The number of fully sequenced genomes and (near) complete genome assemblies is steadily growing (https://en.wikipedia.org/wiki/List_of_sequenced_eukaryotic_genomes; includes links to lists of sequenced archaeal and bacterial genomes).

### Analyses of inserts of known or partially known origin (ISK-2 and ISK-3 scenarios)

Characterization of a newly developed GMO by the developer usually corresponds to an ISK-2 scenario, where the transformation vector sequence is known but the insertion locus or the number of inserts (copy number) is not. Insertion of a recombinant sequence in a functional gene can inactivate the gene and have negative effects on the host under some conditions. Expression of the inserted gene can be influenced by position (i.e. where on a chromosome the gene is inserted [55]). Zhang et al. [56] and Kovalic et al. [57] were the first to provide comprehensive, detailed reports on the characterization of transgene inserts by use of WGS of an animal (cow) and a plant (soybean) respectively. Both performed paired-end sequencing with an Illumina HiSeq sequencer, resulting in the generation of hundreds of millions of 100-bp reads.

In both studies, the reads were mapped against two types of reference sequences to identify the number of inserted copies and their locations in the recipient genomes: (1) the transgenic vector(s) used to transfect/transform the GMO (ISK-2 scenario) and (2) the native recipient genome. This made it possible to identify and characterize completely the inserts and associated event-specific junction sequence motifs. Chimeric reads mapping partially to both types of reference sequence were identified and concatenated (assembled) with overlapping reads into putatively complete junction–insert–junction maps. The inferred maps were successively confirmed by conventional PCR and sequencing of the amplification products. Some rearrangements of the T-DNA relative to the original transgenic vectors were also detected as a result of these approaches.

Zhang et. al. [56] used Illumina HiSeq 2000 sequencing to characterize the number of inserts and the insert locus in three transgenic cows modified to produce recombinant human lactoferrin. They obtained between 246 million and 307 million reads per cow. Previous attempts using chromosome walking analysis had not been successful, possibly because of the large size of the insert (150 kbp) and because multiple copies were inserted. The HTS reads were mapped to the transgenic vector sequence used to transfect/transform the GMO and the bovine reference genome respectively with use of BWA [58]. Zhang et al. observed that sequencing depth analysis was very useful to detect copy number variations of inserted sequence elements, and used this information to more specifically trace and characterize rearrangements (recombinations) of T-DNA. Analysis of sequencing depth over the transformation vectors and the bovine reference genome indicated that the insert could be present in more than one copy. Chimeric paired-end reads and split-reads mapping to both types of reference sequence were analyzed, and the results indicated that six copies were inserted in the same locus at chromosome 5. This result was achieved despite a relatively low average sequencing depth (ten times coverage). The sequence of the junctions between the host and the insert were determined by de novo assembly of chimeric and split reads with use of SOAPdenovo [59]. However, the entire inserted sequence was not reported, and long reads might be required to resolve the repeated structure of the insert. By design and application of a PCR targeting the preinsertion locus, they were also able to discriminate between heterozygous and homozygous transgenes [56].

Kovalic et al. [57] analyzed two representative transgenic soybean lines to determine the number of insertion sites, the insert copy number at each site, and the sequence of each insert and host flanking region. Paired-end sequencing (2 × 100-bp reads) was done with an Illumina HiSeq sequencer, yielding approximately 75 times average sequencing depth with both lines. Kovalic et al. compared their findings with data obtained with the classic method for transgene characterization (i.e., Southern blots). One of the lines was transformed with two different T-DNAs. The soy genome was subtracted by use of BLAST [51], and all surviving reads were re-paired with their mate to capture junction sequences. The specific junction points were identified with use of a custom-developed Perl script. The reads were downsized and only the 5′ part was used for downstream analysis (3–42 bp out of a read length of 100). The selected reads were mapped to the three transformation plasmid sequences, and the locations where mapping ended were taken as potential integration points. The reads were then sorted on the basis of the nonmatching part to determine the number of inserts and the host sequence flanking each insert. The event transformed with two T-DNAs contained a single-locus in vivo rearranged insert. This was correctly identified and characterized, including the junction between the two rearranged T-DNAs of the insert. Not surprisingly it was concluded that HTS-based analysis was simpler and more efficient than a Southern blot approach. The total cost of the characterization was estimated to be less than 50 % of that for Southern blotting. Several advantages over the classic method were highlighted, including consistent experimental design across different constructs, events, and species, and experimentally simpler protocols [57].

In the previously mentioned study by Kawakatsu et al. [25] the transcriptomic data were complemented with paired-end (450–500-bp libraries) WGS performed with an Illumina HiSeq 2000 instrument. An average of approximately 250 million 100-bp reads were obtained for each sample, including a third rice line that had been subjected to various types of mutagenesis and successively cross-bred to produce the a123 line. Reads were mapped to the Nipponbare rice reference genome and the *Agrobacterium* C58 genome with use of CLC Genomics Workbench 5.1, and the T-DNA insertion locus was identified. Structural variants were detected, verified, and characterized by PCR and sequencing. Their transcriptome analysis provided less detailed information for the transgene characterization, as the main focus was to decipher genomic discrepancy between GM crops and their parental lines. Kawakatsu et al. concluded that the pattern of SNPs in the transgenic rice line OSCR11 relative to its parental line a123 was comparable to the pattern that would result from somaclonal variation. Furthermore, they proposed combining WGS and transcriptome analyses to assess genome integrity of GMOs.

Wahler et al. [60] applied essentially the same approach as Kovalic et al. [57] for the identification and characterization of the transgenic insert and insertion sites in event LL62 rice, which is not authorized in the EU. This rice event contains a single short (1493-bp) insert. Approximately 172 million paired-end reads of 75 bp were obtained with an Illumina HiSeq sequencer, yielding an average 65 times sequencing coverage of the rice genome. Wahler et al. treated the LL62 rice as partially unknown; that is; only limited details on the DNA sequences of the transgene insert and vector were available before the study (ISK-3 scenario). More specifically, they started out with the assumption that the applied cloning vector most likely contained one or more of the elements found in commonly used plant transformation vectors such as pCAMBIA-1300. The reads were mapped with an Illumina genome analysis system (Genome Analyzer and CASAVA) against the published rice genome, and breakpoint reads were identified. To screen for putative GM-derived insertions, they assumed that GM-derived inserts would generally be larger than 100 bp. Thus only breakpoints indicating insertions of this size were analyzed further. To distinguish between natural insertions and GM insertions, all breakpoint border sequences of appropriate size were mapped against the pCAMBIA-1300 vector sequence. This approach resulted in the identification of two border sequences belonging to the same breakpoint. Paired-end orphan reads (reads that did not map to the rice genome) were assembled de novo in an iterative process extending the breakpoint sequence into the insert. With seven iterations, the complete single insert was mapped.

Ji et al. [61] wanted to characterize the genomic integration site(s) of the *Pmel-1* mouse, a transgenic model generated with use of two transgenic vectors, containing variable domains of the endogenous T-cell receptor (TCR). The highly identical and repetitive nature of transgenic TCR α and β chains with the endogenous loci and the large size of the construction vectors made it especially challenging to determine the integration site with established methods such as PCR, cloning, fluorescence in situ hybridization, and Southern blot analyses. A homozygous *Pmel-1* transgene mouse was sequenced to eight times coverage with an Illumina HiSeq 2000 sequencer with paired-end reads of 101 bp. To identify candidate integration sites they searched for evidence of structural variants such as tandem duplications and translocations on the basis of discordant read pairs. First the reads were aligned to the reference mouse genome with use of Bowtie [62, 63] in global (end-to-end reads alignment) and local (soft clipped reads) modes, with the software programs DELLY and SVDetect [64, 65]. Unique and discordantly mapped reads were further analyzed to detect structural variants after filtering to remove repeats, mitochondria, centromeres, and intrachromosomal rearrangements. Structural rearrangements were detected with SVDetect, and top scoring read pairs were reviewed and filtered manually to remove structural rearrangements with too few or many supporting reads, structural rearrangements mapping to centromeres or the mitochondrial chromosome, and structural rearrangements with multiple adjacent rearrangements. After the structural variants had been filtered, candidates were ranked on the basis of confidence scores (number of supporting split reads) and the two top candidate duplications associated with TCR regions were identified for further detailed characterization. This resulted in the identification of a potential integration site of the TCR β chain that was subsequently confirmed by PCR analysis. Their approach resembles the analysis in the case of an ISK-3 intragenic scenario.

Srivastava et al. [66], also focusing on transgenic mouse strains, found that transgene inserts are often located in genomic regions that are rich in repetitive sequences such as long and short interspersed nuclear elements. This, in turn, negatively impacts the percentage of uniquely mapping reads around insertion sites. Enrichment for sequences in and around a chromosomal rearrangement can be an effective strategy for solving the signal-to-noise ratio problem but requires custom solutions that easily become cost-prohibitive. Srivastava et al. applied Illumina paired-end sequencing (approximately 18 times coverage, 100-bp reads) and mate-pair sequencing (approximately 32 times coverage; 3–5-kbp fragment size libraries) and demonstrated that identification of integration sites from mate-pair data had high signal-to-noise ratios (examples were improved tenfold) compared with similar analysis on typical short fragment paired-end libraries. Furthermore, they concluded that the low signal-to-noise ratio problem is exacerbated when the insert contains intragenic or cisgenic sequences. The approach they took was based on a priori knowledge of and mapping of reads to the transgene sequence (ISK-2 scenario). However, the transgene sequences were reconstructed in silico on the basis of the cDNA, vector, and/or genomic sequences before bioinformatics analysis. Reads were mapped to transgene sequences with use of the Bowtie 2 short read aligner [62]. Orphaned reads were extracted by means of custom Perl scripts, and were mapped to the mouse genome. Mapping coordinates were used to calculate a distance metric called the “distance to next” (DTN) read. The DTN measures and mapping coordinates to the host and transgene sequence were used to remove PCR duplicates and poor-quality reads. The mouse genome was divided into approximately 300,000 blocks 1 kbp in length, and the scoring scheme aimed at identifying blocks with large numbers of mapped reads with small DTN among them (i.e., the signature of a transgene insertion site).

Endo et al. [17] characterized a mutant rice generated by an *Agrobacterium*-mediated gene targeting technique. This is an approach where the modification occurs through homologous recombination, and should be very precise and not involve the insertion of DNA fragments from *Agrobacterium*. The focus of Endo et al. was to verify the presence/absence of *Agrobacterium*-derived sequences by WGS. Approximately 27 million to 56 million short (average 72-bp) reads (corresponding to five to ten times coverage of the rice genome) were obtained from four gene-targeted plants with an Illumina Genome Analyzer IIx sequencer. The reads were mapped against the *Agrobacterium tumefaciens* C58 genomic DNA, C58 plasmid, and Ti plasmid, and the binary vector used for the gene targeting modification of two of the rice lines with BLAST. Vector-derived sequences were detected only in the two rice lines modified with the vector. No *Agrobacterium* or plasmid sequence was detected in any of the four rice lines. Evaluation of SNPs and short insertions/deletions was done by the mapping of short reads to the Nipponbare rice genome with use of BWA [58]. More than 12,000 variants were called in each of the four samples, corresponding to the difference between the two Nipponbare lines studied (10,575 SNPs and 1556 insertions/deletions).

Guttikonda et al. [67] characterized two soybean events (TE1 and TE2) and their hybrid stack (TE1 × TE2) taking the same approach as Kovalic et al. [57]. A paired-end sequencing library (insert size 800 bp) was prepared from homozygous and hemizygous plant materials and sequenced with an Illumina HiSeq 2000 instrument, yielding 100-bp reads. The WGS was complemented with targeted capture sequencing (discussed later), Southern blot analysis, and Sanger sequencing. The total number of paired-end reads per sample ranged between 95 million and 119 million for the single events (corresponding to ten times coverage of the soybean genome) and up to 147 million for the stack (14 times coverage). Reads were mapped to the soybean Williams82 reference genome and transgene vector sequences with use of BWA [58] and SAMtools [68]. Custom scripts were used to extract the host–insert junctions. With similar amounts of genomic DNA per library the hemizygous material yielded approximately 50 % fewer reads mapping to the T-DNA than the homozygous material (approximately 500 reads vs 1000 reads, corresponding to five times vs ten times coverage), as would be expected. There were three to eight split (chimeric) reads corresponding to junctions between the host and the insert for each of the junctions in the homozygous samples, the respective numbers in the hemizygous (TE2) samples were one or two, and all junctions were identified in each experiment. The inserts were mapped to insertion loci on chromosome 2 (TE2) and chromosome 6 (TE1) of soybean. Discordantly mapped reads spanning the 3′ insert junctions of TE1 and TE2 respectively mapped to the genomic insertion loci of both TE1 and TE2. A common terminator is present in the 3′ region of the two transgenes. No rearrangements of the transgenes were observed with any of the approaches taken, and no vector backbone was detected. However, some rearrangements were observed at the insertion loci of both events.

### Detection and identification of unknown genetic modifications (ISK-4 scenarios)

Yang et al. [69] sequenced two transgenic rice events with an Illumina HiSeq instrument and obtained approximately 100 million paired-end reads of 90 bp, corresponding to an average 25 times sequencing coverage of the rice genome for both events. The data analysis was performed in three different ways, each adapted to a defined a priori insert knowledge scenario. For each scenario, a specific bioinformatics module was designed, with general applicability for transgenic events under similar insert knowledge conditions. Module 1 was intended for ISK-2 scenarios (i.e., the transformation vector and T-DNA construct sequence is known in advance and the sequencing reads can be mapped back to the original vector and T-DNA construct). Each read was mapped independently to the plasmid and the rice genome with BWA [58]. Split reads and discordant reads were extracted and used to infer the insert junctions. This approach is therefore highly comparable to the approaches taken by Zhang et al. [56] and Kovalic et al. [57]. Module 2 was intended for scenarios where only a limited part of the T-DNA construct sequence is known or can be expected to be included in available sequence databases (ISK-3 scenarios). This approach is therefore to some extent comparable to the approach taken by Wahler et al. [60]. Each read was mapped to a database containing 134 known transgenic elements. Reads that matched the database were extracted and de novo assembled with ABySS [70] and the resulting contigs were analyzed with BLAST [51]. Module 3 was intended for ISK-4 scenarios (i.e., when the T-DNA construct sequence is completely unknown and not included in available sequence databases). This module applied an initial genome subtraction strategy: first the reads were mapped to the rice genome for subtraction of hits, before de novo assembly and BLAST analysis of the retained reads. With module 1, two complete inserts with detailed insertion sites were inferred and later verified with PCR and amplicon sequencing for both rice events. With module 2, only one almost correct complete insert with detailed insertions sites were inferred from each of the rice events. With module 3, one almost correct complete insert was inferred from each of the rice events, and one insertion site for one event was also correctly inferred. The study of Yang et al. [69] was consequently the first to describe an approach for detection and characterization of completely unknown transgenic events by use of next-generation WGS. Coverage depth plots presented in supplementary material for that study also indicate that the inserted target copy numbers can be correctly estimated once a putative complete transgenic construction is obtained. The same original sequence data were analyzed with each of the three modules, and an iterative use of modules was foreseen by Yang et al. [69], starting with module 3 (module 2) and using the inferred map as a basis for reanalysis with module 2 (module 1).

In another study, a combined WGS and bioinformatics approach to identification of phylogenomic relationships and unexpected (genes out of the core) data was developed for rapid high-resolution diagnostic typing of single strains/isolates of microorganisms [71]. The specific study focused on potential microbial bioterrorism organisms, including organisms with GM genomes, and was implemented as a Perl script tested in a Linux environment. Instead of genome assembly and mapping to identify orthologous genes, the sequence reads are mapped to identify orthologous sequence reads and calculate the average similarity to characterized core genomes. In short, a window of a defined size was used to select fragments from a sequence read set or moved in steps over a query genome (partially or fully assembled). BLASTN scores between the fragments and a reference genome were collected, because such scores take into account both sequence similarity and the length of hits. Since all fragments were of exactly the same length, the scores were directly comparable. Plotting of scores in a histogram typically yielded a conserved (core) peak and a nonconserved peak. The analyses were based on Roche 454 sequence data, and window sizes of up to 1000 bp were tested. Sequence lengths of 100–200 bp were reported to be most efficient, and thus data from, for example, an Illumina MiSeq system would appear highly suited. To detect genetic modifications, nonconserved reads can be sorted into a separate FASTA file. This file, in turn, can be analyzed further by the mapping of reads to reference database sequences or de novo assembly, detection of putative open reading frames, etc. It is not clear how well the approach would perform with larger genomes and lower genomic divergence than that found in bacteria.

### Capture enrichment from WGS libraries

This section reviews only enrichment approaches applied after creation of a WGS library. For a more comprehensive review of enrichment approaches applied in combination with HTS, see Arulandhu et al. (this volume).

DuBose et al. [72] applied a combination of the Illumina WGS approach with microarray hybridization capture to enrich for the transgene inserts and integration sites and successively identify these. Their approach relies on prior knowledge of the nature and origin of the novel inserted sequences and is consequently only applicable to ISK-2 and ISK-3 scenarios. Similarly to the approaches described by Zhang et al. [56] and Kovalic et al. [57] a library of short genomic DNA fragments suitable for Illumina paired-end sequencing were prepared. Instead of directly sequencing the library of DNA fragments, they first hybridized the library to a custom-designed microarray with probes corresponding to the transgenic vector (a BAC clone). Nonhybridizing fragments were washed off, resulting in 3000–26,000 times enrichment for complete or partial BAC-derived fragments. These were successively eluted and paired-end sequenced with an Illumina HiSeq sequencer, yielding 197 million reads with an average coverage of 82,186 in the enriched area. Reads were mapped to the BAC vector and mouse genome. Regions with breakpoints were identified by SAMtools [68]. Around the four identified BAC/mouse junctions, 8118 discordant read pairs were detected (one read in the BAC and the other in the mouse genome). Three additional unexpected junctions were detected between noncontiguous regions of the BAC, and two large deletions in the BAC (2.1 kbp and 31.7 kbp) were also detected, together with deletions of 90 bp and 1090 bp respectively at the sites of integration in the mouse genome. The applied enrichment approach results in a bias, significantly limiting the range of transgenic sequence motifs that can be detected. It is not truly a WGS approach, but because it involves the initial creation of a whole-genome-derived sequence library, we consider it relevant for the present review. DuBose et al. [72] applied conventional PCR and sequencing of amplification products to verify the putative insertion sites. One of the advantages of their approach, they concluded, is that the enrichment facilitates identification of isogenically derived inserts (cisgenes and intragenes) that can otherwise be very difficult to identify.

Lepage et al. [73] took a similar approach to study integration sites in 64 mutant *Arabidopsis* plants. The whole genome library was prepared according to recommendations for Roche 454 sequencing, with an equivalent quantity of DNA from each of the 64 mutants. However, before sequencing they used biotinylated capture probes complementary to the T-DNA ends to enrich T-DNA-sequence-containing fragments. After hybridization the bound targets were recovered by use of magnetic beads. More than 40 % of the 115,000 reads obtained from the 454 GS FLX sequencer mapped to the T-DNA, and approximately 4000 chimeric reads partially mapped to the T-DNA and partially to the *Arabidopsis* genome with use of the gsMapper module of Newbler version 2.5.3 (454 Life Sciences). Candidate insertion sites were identified and a two-dimensional pooled PCR approach was taken to identify which of the 64 mutants contained each of the candidates. Altogether they managed to locate the T-DNA insertion sites in 55 of the 64 plants.

Another related approach, referred to as “Southern-by-Sequencing” was taken by Zastrow-Hayes et al. [74]. Initially, a capture-probe library was created from approximately 117 kbp of sequence from a plasmid pool of 89 unique transformation plasmids. The resulting biotinylated oligonucleotide capture probes were then hybridized to Illumina WGS libraries for enrichment of plasmid-derived sequences and neighboring sequences. The enriched libraries were successively sequenced with an Illumina MiSeq or HiSeq 2500 system, yielding paired-end 100-bp reads to a target coverage of approximately 100 times. Sequencing errors were filtered by *K*-mer analysis with JELLYFISH [75], and identical sequence reads were collapsed to create nonredundant read groups. Nonredundant reads were then aligned to the reference genome (maize) with use of Bowtie [63], and reads that did not align to the reference genome were subsequently aligned to the transformation plasmid insert and backbone sequences with use of Bowtie 2 [62]. Alignments to the backbone sequence with 35 times or higher coverage across 50 bp or more were flagged as events containing plasmid backbone sequence. This reduced the rate of false positives due to environmental bacteria. Reads that did not map entirely to the maize genome were subjected to junction analysis with BWA [76] to map reads against the plasmid insert and backbone sequences. Split reads (i.e., chimeric reads mapping partly to the transformation plasmids) were retained after filtering, and compared with reads from non-GM lines to remove endogenous junctions (i.e., junctions of non-GM origin present as natural variants). The final junctions were then assembled into longer contigs with use of SSAKE [77]. Subsequently the longest SSAKE contigs were mapped to the (maize) reference genome and the transformation plasmids to characterize the insertion sites and intactness of the inserted DNA with use of BLAT [78]. Full assembly of the entire T-DNA insertions was not possible with the procedures described but was predicted to become feasible with additional advances in sequencing technologies and sample preparation methods.

The previously mentioned study by Guttikonda et al. [67] included use of target capture sequencing as a complement to WGS, Southern blot analysis, and Sanger sequencing. For this, they used their original WGS libraries, and the approach was similar to that of DuBose et al. [72], except that the custom probes were coupled to beads instead of a microarray. Unbound fragments were washed away and PCR was successively performed to enrich libraries attached to the capture beads. The resulting enriched libraries were then paired-end sequenced with an Illumina MiSeq system, yielding 250-bp reads. The capture enrichment approach yielded a 250–2000 times improvement in coverage of the insert and junctions.

### Whole genome HTS-based screening analyses for detection and identification of GMOs and derived products

Willems et al. [79] estimated the feasibility and required size of sequence datasets from HTS for routine analysis of GM crops. They distinguished between three approaches, termed the “detection approach,” “proof approach,” and “identification approach.” “Detection” corresponded to one or more reads mapping entirely to the insert, “proof of integration” corresponded to one or more mate pairs with one read mapping entirely to the insert and the other mapping entirely to the host/recipient genome, and “identification” corresponded to one or more chimeric reads mapping partly to the insert and partly to the host/recipient genome. Not surprisingly, they identified the genome size as the most influential parameter. Several parameters in their statistical framework are defined by the user, such as the desired probability of detection and the definition of the required number of overlapping base pairs for scoring a hit with the identification approach. They concluded that all three approaches would require less than or the same as the output of a single lane on an Illumina rapid run (300 million reads) with any type of 100 % GM sample. However, proof and identification would require severalfold higher sequence output than detection, and samples containing only low fractions of GMO (e.g., 0.9 %, the threshold for labeling in the EU [80]), would in most cases require prohibitively large datasets (given the cost and output of current HTS technologies). The statistical framework was experimentally validated with 100 % pure and processed rice samples and an in-house-prepared 10 % GM in non-GM rice sample.

Experimental data from HTS of a commercial maize gluten feed sample from the USA (TAME3; unpublished data) largely support the validity of the statistical framework of Willems et al. [79]. Approximately 228.6 million paired-end reads (read length 100 bp) corresponding to 45.7 Gbp, were obtained from this highly processed product (fragment size range of purified DNA approximately 100–500 bp). Because of the short fragment size, the paired-end reads frequently overlapped, and were consequently stitched before mapping. After stitching and quality filtering, approximately 224 million reads (weighted mean length 124 bp) were retained. Approximately 61.1 % of the reads (126.5 million) mapped to the assembled maize genome (haploid genome size excluding chloroplast and mitochondrial genomes 2.07 Gbp [7]), corresponding to a mean coverage of approximately 7.58 times the entire maize genome (*C* = *N* × *L*/*S* = 126.5 × 10^6^ × 124/2.07 × 10^9^ = 7.58, where *C* is coverage, *N* is the number of reads, *L* is the mean length of a read, and *S* is the number of base pairs in the haploid maize genome). In the EU, according to the GMO labeling regulation [80], the GMO concentration is calculated per ingredient; that is, as the additive concentration of all EU-authorized events belonging to the ingredient (species). In line with this and on the basis of event-specific quantitative real-time (qPCR), it was estimated that the feed contained approximately 200 % EU-authorized GM maize [expressed in copy number ratio of GM/endogenous gene (*hmg1*)]. This was due to the presence of ten different events at individual concentrations ranging from 3.2 % (MON863) to 38.2 % (DAS1507). One additional EU-authorized GM maize event was detected at a concentration below the limit of quantification (Table 3). A concentration greater than 100 % implies that at least some of the GM maize in this sample must be derived from hybrid stacks of authorized events. If we apply the terminology of Willems et al. [79], the corresponding quantity determined from HTS-based “identification” of the same target motifs as those detected by qPCR was 233 % (Table 4). The observed number of hits for both the maize reference gene and the event-specific target motifs was approximately 50 % of that predicted from the coverage rate and qPCR data (Table 4). It is possible that this is due to stochastic variation given the low mean coverage predicted. However, according to Table 3 of Willems et al. [79], the expected number of sequenced reads required to obtain at least one “identification” read in a 100 % GM maize sample is approximately 42 million. The sample was estimated to be 200 % GM by qPCR, and consequently it was predicted that approximately six hits were to be expected from the 126.5 million filtered reads, corresponding well with the observed seven hits (Table 4). A more systematic and comprehensive analysis of the data, including the full length of inserts and multiple genomic loci in maize, is currently ongoing (Spilsberg et al., unpublished).

**Table 3:** Identity and quantity of European Union (EU)-authorized genetically modified organisms (GMOs) detected by event-specific quantitative real-time polymerase chain reaction (qPCR) in a commercial maize gluten feed sample from the USA

GMO event^a^	OECD UI [81]	Measured concentration^b^	Target^c^	Amplicon size (bp)	Reference^d^
1507 maize	DAS-Ø15Ø7-1	38.2 %	3′ junction	58	QT-EVE-ZM-010
MON88017 maize	MON-88Ø17-3	34.0 %	3′ junction	95	QT-EVE-ZM-016
MON810 maize	MON-ØØ81Ø-6	32.0 %	5′ junction	92	QT-EVE-ZM-020
59122 maize	DAS-59122-7	31.7 %	5′ junction	86	QT-EVE-ZM-012
NK603 maize	MON-ØØ6Ø3-6	27.5 %	3′ junction	108	QT-EVE-ZM-008
Bt11 maize	SYN-BTØ11-1	11.6 %	Construct^e^	75	[82]
MIR604 maize	SYN-IR6Ø4-5	10.1 %	5′ junction	76	QT-EVE-ZM-013
GA21 maize	MON-ØØØ21-9	5.9 %	5′ junction	101	QT-EVE-ZM-014
MON89034 maize	MON-89Ø34-3	4.1 %	3′ junction	77	QT-EVE-ZM-018
MON863 maize	MON-ØØ863-5	3.2 %	5′ junction	84	QT-EVE-ZM-009
T25 maize	ACS-ZMØØ3-2	<LOQ	3′ junction	102	QT-EVE-ZM-011
MIR162	SYN-IR162-4	ND	3′ junction	92	QT-EVE-ZM-022
MON87460 maize	MON 8746Ø-4	ND	5′ junction	82	QT-EVE-ZM-005
Maize total	NA	198.3 %	NA	58–108	NA

*LOQ* limit of quantification, *NA* not available, *ND* not detected, *OECD* Organisation for Economic Co-operation and Development, *UI* unique identifier

^a^Commonly used names. Other names are frequently used in commercial trade. This list includes all genetically modified maize events authorized in the EU as of November 1, 2015.

^b^Concentration measured on the basis of qPCR standard curves obtained with certified reference materials of the GMO events and the endogenous single copy reference gene *hmg1* in maize (amplicon size 79 bp, QT-TAX-ZM-002 [83])

^c^All qPCRs used are under ISO17025 accreditation at the National Institute of Biology, Slovenia.

^d^The numbers refer to specific modules in the collection of validated qPCR methods in the EU’s Reference Laboratory for Genetically Modified Food and Feed methods database [83] or the cited publication.

^e^For this event, a specific, validated construct-specific qPCR was used.

**Table 4:** Identity and quantity of EU-authorized GMOs from high-throughput sequencing (*HTS*) -based screening analysis of a commercial maize gluten feed sample from the USA (TAME3)

GMO event^a^	Expected no. of hits^b^	Observed no. of hits^c^	HTS-based GMO concentration observed^d^	Concentration measured by qPCR^e^	Target^e^
1507 maize	2.90/1.15	1	33 %	38.2 %	3′ junction
MON88017 maize	2.58/1.02	0	0 %	34.0 %	3′ junction
MON810 maize	2.43/0.96	1	33 %	32.0 %	5′ junction
59122 maize	2.40/0.95	1	33 %	31.7 %	5′ junction
NK603 maize	2.08/0.83	2	67 %	27.5 %	3′ junction
Bt11 maize	0.88/0.35	2	67 %	11.6 %	Construct
MIR604 maize	0.77/0.30	0	0 %	10.1 %	5′ junction
GA21 maize	0.45/0.18	0	0 %	5.9 %	5′ junction
MON89034 maize	0.31/0.12	0	0 %	4.1 %	3′ junction
MON863 maize	0.24/0.10	0	0 %	3.2 %	5′ junction
T25 maize	<0.01/<0.01	0	0 %	<LOQ	3′ junction
MIR162	0/0	0	0 %	ND	3′ junction
MON87460 maize	0/0	0	0 %	ND	5′ junction
Maize total	15.03/5.95	7	233 %	198.3 %	NA
Maize *hmg1* reference gene	7.58/NA	3	NA (100 %)	NA (100 %)	Slightly expanded qPCR motif

^a^See Table 3 for more details on the identity of specific events.

^b^ *A*/*B*, where *A* is the estimated number of hits based on the mean coverage across the maize genome (7.58 times) and *B* is the estimated number of hits based on the observed hits for the reference gene by HTS (maize *hmg1*; last row in the table).The estimated number of hits is *R* × *Q*/100, where *R* is the number of haploid maize genome copies and *Q* is the concentration (%) detected by qPCR (see Table 3).

^c^Only the number of “identification” hits is given. Any number greater than zero means that the specific junction motif detected by qPCR was detected by HTS.

^d^Calculated according to the equation GM% = cpGM × 100/cpREF, where cpGM is the observed number of copies of the genetically modified target and cpREF is the observed number of copies of the reference gene (here maize *hmg1*)

^e^See Table 3. HTS mapping of reads was done against one reference sequence per qPCR target. A minimum overlap across the event-specific junction of 5 bp was required. With a maximum read length of 100 bp, the HTS mapping targeted up to 2 × 95 bp of sequence.

## Discussion

Detailed information about the modified nucleic acids found in GMOs is important for several stakeholders. The developers of GMOs need to know their products to be able to evaluate them (e.g., for further selection and testing) and to provide legally and contractually required information to other stakeholders. Public risk assessors, for example, members of the European Food Safety Authority Panel on Genetically Modified Organisms, use the data to assess the safety of GMOs with regard to health and the environment [30, 84, 85]. The food industry and public and private control laboratories use the data to develop and validate detection methods and for surveillance and monitoring of the distribution of GMOs and derived materials.

**Fig. 3:**
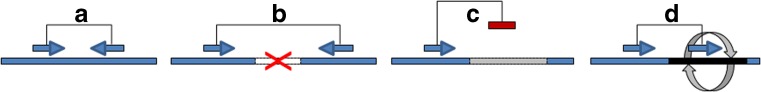
Sensitivity of paired-end sequencing to rearrangements when mapping is done to a reference sequence. **a** Normal paired-end reads have opposite orientation and the distance between them is predicted by the fragment size used to create the sequencing library (e.g., 500 bp). **b** A deletion (e.g., 200 bp) in the target relative to the reference sequence will map the two reads to more distant positions than those predicted from the sequencing library (e.g., 700 bp instead of 500 bp). **c** An insertion in the target relative to the reference sequence will result in discordant mapping of one of the reads. With sufficiently high coverage, there will be a significantly increased density of discordantly mapped reads adjacent to the insertion. **d** Inversion of a part of the target sequence relative to the reference will reorient one of the mate reads, resulting in mapping of both reads in the same orientation

Developers of GMOs will normally have access to detailed information about the vectors and T-DNA inserts before the creation of the GMO. They also have access to the non-GM isogenic line or strain that received the T-DNA. And finally, with very few exceptions, genome sequencing projects targeting the species subject to genetic modification are usually either completed or ongoing, facilitating access to sequenced reference genomes. Intraspecific variation can be an obstacle but generally these genome sequences provide solid foundations for successful detection and characterization of T-DNA inserts and insertion sites in GMOs by comparative resequencing approaches. The observable differences between the WGS of the GMO and its isogenic non-GM counterpart hold key information not only regarding the inserts but also regarding possible unintended genomic rearrangements that may or may not be related to the genetic modification as such. Knowledge of the vector and the intended T-DNA insert provides sequence tags that can be used either for direct comparison or for a genome walking assembly strategy to unravel the complete inserts and insertion sites. The read length can have a very significant impact on the ability to infer correct genetic maps of transgene inserts, insertion loci, and rearrangements. The earliest dominating HTS technology (Roche 454) yielded reads up to 700 bp but at a relatively high price per sequenced million base pairs. Most of the published studies discussed herein applied Illumina technology, typically yielding read lengths of 100 bp, but potentially up to maximum of 250–300 bp. Paired-end and mate-pair sequencing as made available with Illumina technology, however, facilitates detection of small rearrangements in reference sequences (e.g., genomic insertion loci or vectors of T-DNA; Fig. 3) and contig assembly. With recently commercialized platforms such as PacBio and MinION it is possible to obtain very long reads (several thousand base pairs), potentially corresponding to complete transgenic inserts. Combinations of platforms and strategies may prove ideal to obtain detailed and verified information. Older technologies such as PCR and Sanger sequencing, Southern blot, and fluorescence in situ hybridization are also options for verification of the data. Several of the articles cited [45, 57, 61, 67, 69, 74] conclude that the use of WGS offers great advantages over traditionally used approaches, and some of the authors of those articles are major biotechnology developers. It is therefore reasonable to expect that the developers of GMOs will adopt and exploit HTS technology to improve the quality of the data and genetic maps from characterization of GMOs. Whether or not the developers will also make the sequence data available—for example, to risk assessors—may depend on the specific legal requirements in relevant jurisdictions. This again may depend on the perceived relevance of the data.

**Fig. 4:**
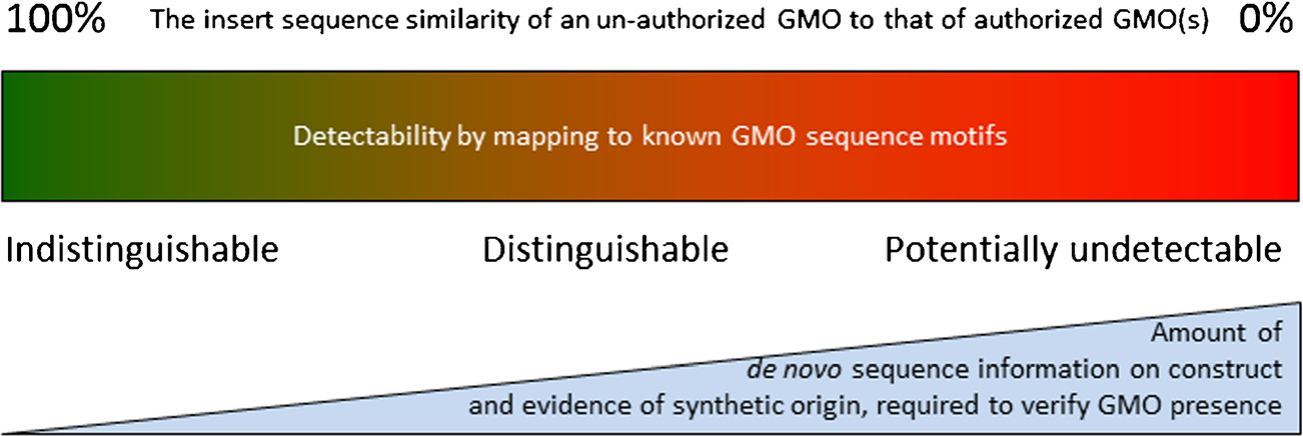
Relevance of similarity to known genetic modifications for discriminatory power and identifiability of unknown and unauthorized genetic modifications. Gene editing technologies such as CRISP–Cas9 can produce GMOs that are nearly indistinguishable from non-GMOs and therefore do not fit well in the figure

Legally, the possibilities to use gene editing technologies such as CRISPR–Cas9 [17–21] represent a paradigm shift for the detectability of GMOs. Traditional GMOs, including those with cisgenes, are characterized by insertions, deletions, and rearrangements of large DNA fragments (typically several thousand base pairs long; Fig. 1). The traditional GMOs are therefore clearly distinguishable from conventional mutants. Contrastingly, gene editing can introduce very small changes, such as SNMs that are indistinguishable from SNPs. Although these changes are detectable by DNA sequencing, it is difficult or even impossible to prove that such a change is the result of the use of gene technology (and therefore by definition a GMO) and not a (naturally derived) mutation/substitution (see Fig. 4). Disclosure of information on the specific modifications from the developers will be a necessity. Linking the SNM with other authenticating genetic signatures [86] present in the GMO could perhaps provide sufficient proof, at least in some cases. Identifying a GM origin of a product of gene editing is not the only type of borderline case that challenges GMO detection laboratories and authorities. The definition of GMOs in the EU, for example, is under revision, and the possibility to include or exclude products (i.e., living organisms) that contain no modified nucleic acid is being debated [19].

Public risk assessors will usually receive detailed information from the developing biotechnology company in connection with applications for authorization or deregulation of the GMO for use and/or release into the environment. If so, the level of detail can be very high, as explained above. However, there can be cases where public risk assessors receive the information from other, less well-informed sources. This has, for example, been the case for several unauthorized GMOs that illegally entered the EU food/feed supply chain [45] (reviewed in [27]). A typical case is initiated by the observed presence of a GMO-associated molecular marker in a GMO screening test incompatible with the presence of only authorized GMOs [27]. Further analyses then confirm the presence of an unauthorized GMO-derived sequence motif, reinforcing the suspicion of the presence of an unauthorized GMO. If the GMO is completely risk assessed, then a specific detection method is also available and can be used for the identification of the GMO. However, in the case of an incompletely risk-assessed GMO, such a method is only exceptionally available. The available information forwarded to risk assessors is typically limited to the DNA sequence of an identified novel trait gene and its associated promoter and/or terminator. Further details regarding insertion sites, presence or absence of additional inserts, vector backbone sequence, and/or additional traits are desirable for a comprehensive molecular analysis as part of a risk assessment procedure. The application of HTS technology has the potential to provide this information. Most of the approaches described and discussed herein would fit this purpose. However, in the exceptional cases where suspicion is not based on molecular evidence and no reliable sequence information on a suspected GMO is available (ISK-4), the only approaches described so far that could succeed are de novo assembly and exome sequencing approaches; see [45, 50, 69].

Pauwels et al. [16] discussed the possible contributions from HTS-based molecular characterization of GMOs to risk assessments. Despite the superiority of HTS approaches over more traditional molecular approaches with respect to the amount and quality of data (see their Table 1), Pauwels et al. questioned the added value from these new technologies for risk assessments and pointed out some challenges; for example, the lack of standardized approaches for data generation, and requirements for specialists in bioinformatics to select the appropriate data analysis strategies. This implies that it will be difficult for risk assessors to assess the quality and reliability, and interpret the data. Another point they mention is the relevance of small inserts to the actual risks. Although HTS can provide evidence of such inserts and lead to their characterization, this information may not be needed, taking into account that unintended effects in GM plants are also assessed on the basis of agronomic, phenotypic, and compositional properties. An insert with functional effect on the GM plant would most likely result in an observable phenotypic change. Pauwels et al. concluded that setting up a common workflow for the generation of relevant and interpretable data by HTS would facilitate a scientifically sound assessment of GM plants. It may be argued that the mere availability of as much information as possible is beneficial for any evaluation, including risk assessments. Information overload could, however, also delay and confuse the evaluation in the absence of clear guidelines and requirements for data generation, analysis, and interpretation. Understanding the risk assessment procedure (as outlined for the EU, for example, in [84, 85]) may be required to be able to judge the potential value and limitations of HTS data. Unintended effects can also arise from conventional breeding and natural molecular mechanisms. In this case undesirable phenotypes are removed during selection and breeding programs that do not require molecular characterization or risk assessments.

Screening for the presence of unauthorized GMOs is not possible unless the targets can be identified. Screening tests performed routinely are therefore presently targeted. The more the molecular characteristics of the genetic modification are similar to or distinctive from those of all other GMOs, the likelier it is that the GMO will not be detectable with that approach (Fig. 4). If it is justifiable to invest the necessary resources in further analysis, then the logical solution to this challenge is to perform WGS or exome sequencing and focus the analyses on the sequence motifs that do not match the native (nonmodified) genome or exome. In the future it may be that HTS will become the method of choice also for screening analyses. HTS-based screening tests could be substantially broader and less targeted than those currently applied using PCR techniques [40, 87, 88]. Shotgun-sequencing-based screening approaches may become the most cost-effective alternative if technological developments continue at the pace that has been seen in the last decade: (1) the costs of sequencing will be further and substantially reduced; (2) sequencing speed and throughput will continue to improve; (3) WGS assemblies will be made available for more species; (4) more lines of the sequenced species will be resequenced and the sequences made available; and (5) bioinformatics pipelines will be developed and/or improved for the purpose and become (semi)automated and faster. All of these requirements can be realistically achieved. Combining different sequencing tools as well as libraries of different insert sizes (paired-end sequencing in combination with mate-pair sequencing) may further facilitate efficient and comprehensive identification of GM-derived sequences in complex samples. The major potential of HTS in this respect is not the cost-efficiency but the significantly broader spectrum of GMOs that could be made detectable, contributing to enforcement of the GMO legislation.

The interpretation of analytical results is not trivial; in particular, when a sequence motif is detected and no specific GM source can be identified. This is perhaps best exemplified with PCR-based screening as discussed in [89]. Assembly of short sequence reads into longer contigs, and successive verification of the presence of whole genes, constructs, and eventually insertion sites by PCR and sequencing, has the potential to facilitate interpretation and provide indisputable evidence in the future. The feasibility of this on, for example, processed food or feed samples containing mixtures of several species and/or GMOs mixed with non-GM material is yet to be demonstrated in full. Very high numbers of sequenced base pairs are required to achieve an acceptable limit of detection [79], and the successive bioinformatics analysis would be challenging and require highly skilled personnel. The current sequencing costs and the successive bioinformatics workload renders this too costly and time-consuming to be applicable at present. However, technological developments have reduced the costs and increased the output of sequencing dramatically in the last decade, and (semi)automated bioinformatics pipelines may be developed. Together these developments suggest the feasibility of this type of approach at affordable cost in the not too distant future. This should be communicated and appreciated by all stakeholders, and is perhaps one of the most important added values that can be foreseen from application of WGS for GMO detection. For the plant breeding sector and for particular cases where more or less pure single-GMO samples are available, the technology can already be applied and is expected to provide very detailed and comprehensive molecular data. The application for these purposes is consequently already ongoing.

## References

[1] Sanger F, Nicklen S, Coulson AR (1977). DNA sequencing with chain-terminating inhibitors. Proc Natl Acad Sci U S A.

[2] Lander ES, Linton LM, Birren B, Nusbaum C, Zody MC, International Human Genome Sequencing Consortium (2001). Initial sequencing and analysis of the human genome. Nature.

[3] Waterston RH, Lindblad-Toh K, Birney E, Rogers J, Abril JF, Agarwal P (2002). Initial sequencing and comparative analysis of the mouse genome. Nature.

[4] Matsumoto T, Wu JZ, Kanamori H, Katayose Y, Fujisawa M, Namiki N (2005). The map-based sequence of the rice genome. Nature.

[5] Schmutz J, Cannon SB, Schlueter J, Ma J, Mitros T, Nelson W (2010). Genome sequence of the palaeopolyploid soybean. Nature.

[6] Schadt EE, Turner S, Kasarskis A (2010). A window into third-generation sequencing. Hum Mol Genet.

[7] Schnable PS, Ware D, Fulton RS, Stein JC, Wei F, Pasternak S (2009). The B73 maize genome: complexity, diversity, and dynamics. Science.

[8] Mayer KFX, Rogers J, Dolezel J, Pozniak C, Eversole K, Feuillet C, et al. A chromosome-based draft sequence of the hexaploid bread wheat (*Triticum aestivum*) genome. Science. 2014;345(6194):1251788. doi:10.1126/science.1251788. 10.1126/science.1251788 25035500

[9] Jiang Y, Xie M, Chen W, Talbot R, Maddox JF, Faraut T (2014). The sheep genome illuminates biology of the rumen and lipid metabolism. Science.

[10] Groenen MAM, Archibald AL, Uenishi H, Tuggle CK, Takeuchi Y, Rothschild MF (2012). Analyses of pig genomes provide insight into porcine demography and evolution. Nature.

[11] Zimin AV, Delcher AL, Florea L, Kelley DR, Schatz MC, Puiu D, et al. A whole-genome assembly of the domestic cow, *Bos taurus*. Genome Biology. 2009;10(4):R42. doi:10.1186/gb-2009-10-4-r42. 10.1186/gb-2009-10-4-r42 PMC2688933 19393038

[12] Kim SK, Nair RM, Lee J, Lee S-H (2015). Genomic resources in mungbean for future breeding programs. Front Plant Sci.

[13] Ramakrishnan AP, Ritland CE, Blas Sevillano RH, Riseman A (2015). Review of potato molecular markers to enhance trait selection. Am J Potato Res.

[14] European Commission (2001). Directive 2001/18/EC of the European Parliament and the Council of 12 March 2001 on the deliberate release into the environment of genetically modified organisms and repealing Council Directive 90/220/EEC. Off J Eur Communities L.

[15] Norwegian Government, Ministry of Climate and Environment. Gene Technology Act. Act of 2 April 1993 no. 38 relating to the production and use of genetically modified organisms, etc. (amended 2005). https://www.regjeringen.no/en/dokumenter/gene-technology-act/id173031/. Accessed 5 Feb 2016.

[16] Pauwels K, De Keersmaecker S, De Schrijver A, du Jardin P, Roosens N, Herman P (2015). Next-generation sequencing as a tool for the molecular characterization and risk assessment of genetically modified plants: added value or not?. Trends Food Sci Technol.

[17] Endo M, Kumagai M, Motoyama R, Sasaki-Yamagata H, Mori-Hosokawa S, Hamada M (2015). Whole-genome analysis of herbicide-tolerant mutant rice generated by *Agrobacterium*-mediated gene targeting. Plant Cell Physiol.

[18] Jinek M, Chylinski K, Fonfara I, Hauer M, Doudna JA, Charpentier E (2012). A programmable dual-RNA-guided DNA endonuclease in adaptive bacterial immunity. Science.

[19] Lusser M, Parisi C, Plan D, Rodriguez-Cerezo E. Deployment of new biotechnologies in plant breeding. Nat Biotechnol. 2012;30(3):231–239. doi:10.1038/nbt.2142. 10.1038/nbt.2142 PMC7097357 22398616

[20] Podevin N, Davies HV, Hartung F, Nogue F, Casacuberta JM (2013). Site-directed nucleases: a paradigm shift in predictable, knowledge-based plant breeding. Trends Biotechnol.

[21] Sander JD, Joung JK (2014). CRISPR-Cas systems for editing, regulating and targeting genomes. Nat Biotechnol.

[22] Batista R, Saibo N, Lourenco T, Oliveira MM (2008). Microarray analyses reveal that plant mutagenesis may induce more transcriptomic changes than transgene insertion. Proc Natl Acad Sci U S A.

[23] Gao L, Cao Y, Xia Z, Jiang G, Liu G, Zhang W, et al. Do transgenesis and marker-assisted backcross breeding produce substantially equivalent plants? - A comparative study of transgenic and backcross rice carrying bacterial blight resistant gene Xa21. BMC Genomics. 2013;14:738. doi:10.1186/1471-2164-14-738. 10.1186/1471-2164-14-738 PMC4007521 24165682

[24] Heinemann JA, Kurenbach B, Quist D (2011). Molecular profiling - a tool for addressing emerging gaps in the comparative risk assessment of GMOs. Environ Int.

[25] Kawakatsu T, Kawahara Y, Itoh T, Takaiwa F (2013). A whole-genome analysis of a transgenic rice seed-based edible vaccine against cedar pollen allergy. DNA Res.

[26] Bell CC, Magor GW, Gillinder KR, Perkins AC. A high-throughput screening strategy for detecting CRISPR-Cas9 induced mutations using next-generation sequencing. BMC Genomics. 2014;15:1002. doi:10.1186/1471-2164-15-1002. 10.1186/1471-2164-15-1002 PMC4246457 25409780

[27] Holst-Jensen A, Bertheau Y, de Loose M, Grohmann L, Hamels S, Hougs L (2012). Detecting un-authorized genetically modified organisms (GMOs) and derived materials. Biotechnol Adv.

[28] Neelakandan AK, Wang K (2012). Recent progress in the understanding of tissue culture-induced genome level changes in plants and potential applications. Plant Cell Rep.

[29] Biosafety Clearing House. Cartagena protocol on biosafety to the convention on biological diversity. Biosafety Clearing-House. 2000. http://bch.cbd.int/database/attachment/?id=10694. Accessed 5 Feb 2016.

[30] Codex Alimentarius. Principles for the risk analysis of foods derived from modern biotechnology. CAC/GL 44-2003. 2003. ftp://ftp.fao.org/es/esn/food/princ_gmfoods_en.pdf. Accessed 5 Feb 2016.

[31] Li J-Y, Wang J, Zeigler RS (2014). The 3,000 rice genomes project: new opportunities and challenges for future rice research. Gigascience.

[32] Dong W, Yang L, Shen K, Kim B, Kleter G, Marvin H (2008). GMDD: a database of GMO detection methods. BMC Bioinform.

[33] Benson DA, Clark K, Karsch-Mizrachi I, Lipman DJ, Ostell J, Sayers EW (2015). GenBank. Nucleic Acids Res.

[34] Kyndt T, Quispe D, Zhai H, Jarret R, Ghislain M, Liu Q (2015). The genome of cultivated sweet potato contains *Agrobacterium* T-DNAs with expressed genes: an example of a naturally transgenic food crop. Proc Natl Acad Sci U S A.

[35] Vinner L, Mourier T, Friis-Nielsen J, Gniadecki R, Dybkaer K, Rosenberg J, et al. Investigation of human cancers for retrovirus by low-stringency target enrichment and high-throughput sequencing. Sci Rep. 2015;5:13201. doi:10.1038/srep13201. 10.1038/srep13201 PMC4541070 26285800

[36] Vallenback P, Ghatnekar L, Bengtsson BO (2010). Structure of the natural transgene *PgiC2* in the common grass *Festuca ovina*. PLoS One.

[37] Holst-Jensen A, Berdal K, Bertheau Y, Bohanec M, Bohlin J, Chaouachi M, Bertheau Y (2013). Towards detection of unknown GMOs. Genetically modified and non-genetically modified food supply chains: co-existence and traceability.

[38] Block A, Debode F, Grohmann L, Hulin J, Taverniers I, Kluga L, et al. The GMOseek matrix: a decision support tool for optimizing the detection of genetically modified plants. BMC Bioinform. 2013;14:256. doi:10.1186/1471-2105-14-256. 10.1186/1471-2105-14-256 PMC3765097 23965170

[39] Scholtens I, Laurensse E, Molenaar B, Zaaijer S, Gaballo H, Boleij P (2013). Practical experiences with an extended screening strategy for genetically modified organisms (GMOs) in real-life samples. J Agric Food Chem.

[40] Waiblinger H, Grohmann L, Mankertz J, Engelbert D, Pietsch K (2010). A practical approach to screen for authorised and unauthorised genetically modified plants. Anal Bioanal Chem.

[41] Mayer KFX, Waugh R, Langridge P, Close TJ, Wise RP, Graner A (2012). A physical, genetic and functional sequence assembly of the barley genome. Nature.

[42] Ming R, Hou S, Feng Y, Yu Q, Dionne-Laporte A, Saw JH (2008). The draft genome of the transgenic tropical fruit tree papaya (*Carica papaya* Linnaeus). Nature.

[43] RASFF - food and feed safety alerts. European Commission, Brussels. http://ec.europa.eu/food/safety/rasff/index_en.htm. Accessed 5 Feb 2016.

[44] Boetzer M, Henkel CV, Jansen HJ, Butler D, Pirovano W (2011). Scaffolding pre-assembled contigs using SSPACE. Bioinformatics.

[45] Barbau-Piednoir E, De Keersmaecker SCJ, Wuyts V, Gau C, Pirovano W, Costessi A, et al. Genome sequence of EU-unauthorized genetically modified *Bacillus subtilis* strain 2014-3557 overproducing riboflavin, isolated from a vitamin B2 80% feed additive. Genome Announc. 2015;3(2). doi:10.1128/genomeA.00214-15. 10.1128/genomeA.00214-15 PMC4392148 25858836

[46] Barbau-Piednoir E, de Keersmaecker S, Delvoye M, Gau C, Philipp P, Roosens N (2015). Use of next generation sequencing data to develop a qPCR method for specific detection of EU-unauthorized genetically modified *Bacillus subtilis* overproducing riboflavin. BMC Biotechnol.

[47] Franco-Zorrilla JM, Lopez-Vidriero I, Carrasco JL, Godoy M, Vera P, Solano R (2014). DNA-binding specificities of plant transcription factors and their potential to define target genes. Proc Natl Acad Sci U S A.

[48] Hernandez-Garcia CM, Finer JJ (2014). Identification and validation of promoters and cis-acting regulatory elements. Plant Sci.

[49] Zabidi MA, Arnold CD, Schernhuber K, Pagani M, Rath M, Frank O (2015). Enhancer-core-promoter specificity separates developmental and housekeeping gene regulation. Nature.

[50] Tengs T, Zhang H, Holst-Jensen A, Bohlin J, Butenko M, Kristoffersen A (2009). Characterization of unknown genetic modifications using high throughput sequencing and computational subtraction. BMC Biotechnol.

[51] Altschul SF, Madden TL, Schaffer AA, Zhang JH, Zhang Z, Miller W (1997). Gapped BLAST and PSI-BLAST: a new generation of protein database search programs. Nucleic Acids Res.

[52] Oh S, Song S, Kim Y, Jang H, Kim S, Kim M (2005). *Arabidopsis* CBF3/DREB1A and ABF3 in transgenic rice increased tolerance to abiotic stress without stunting growth. Plant Physiol.

[53] Ouyang S, Zhu W, Hamilton J, Lin H, Campbell M, Childs K (2007). The TIGR rice genome annotation resource: improvements and new features. Nucleic Acids Res.

[54] Anders S, Huber W. Differential expression analysis for sequence count data. Genome Biol. 2010;11(10):R106. doi:10.1186/gb-2010-11-10-r106. 10.1186/gb-2010-11-10-r106 PMC3218662 20979621

[55] Garrick D, Fiering S, Martin DIK, Whitelaw E (1998). Repeat-induced gene silencing in mammals. Nat Genet.

[56] Zhang R, Yin Y, Zhang Y, Li K, Zhu H, Gong Q, et al. Molecular characterization of transgene integration by next-generation sequencing in transgenic cattle. PLoS One. 2012;7(11):e50348. doi:10.1371/journal.pone.0050348. 10.1371/journal.pone.0050348 PMC3503979 23185606

[57] Kovalic D, Garnaat C, Guo L, Yan Y, Groat J, Silvanovich A (2012). The use of next generation sequencing and junction sequence analysis bioinformatics to achieve molecular characterization of crops improved through modern biotechnology. Plant Genome.

[58] Li H, Durbin R (2009). Fast and accurate short read alignment with Burrows-Wheeler transform. Bioinformatics.

[59] Luo R, Liu B, Xie Y, Li Z, Huang W, Yuan J, et al. SOAPdenovo2: an empirically improved memory-efficient short-read de novo assembler. Gigascience. 2012;1(1):18. doi:10.1186/2047-217x-1-18. 10.1186/2047-217X-1-18 PMC3626529 23587118

[60] Wahler D, Schauser L, Bendiek J, Grohmann L (2013). Next-generation sequencing as a tool for detailed molecular characterisation of genomic insertions and flanking regions in genetically modified plants: a pilot study using a rice event unauthorised in the EU. Food Anal Methods.

[61] Ji Y, Abrams N, Zhu W, Salinas E, Yu Z, Palmer DC, et al. Identification of the genomic insertion site of *Pmel-1* TCR alpha and beta transgenes by next-generation sequencing. PLoS One. 2014;9(5):e96650. doi:10.1371/journal.pone.0096650. 10.1371/journal.pone.0096650 PMC4020793 24827921

[62] Langmead B, Salzberg SL (2012). Fast gapped-read alignment with Bowtie 2. Nat Methods.

[63] Langmead B, Trapnell C, Pop M, Salzberg SL. Ultrafast and memory-efficient alignment of short DNA sequences to the human genome. Genome Biol. 2009;10(3):R25. doi:10.1186/gb-2009-10-3-r25. 10.1186/gb-2009-10-3-r25 PMC2690996 19261174

[64] Rausch T, Zichner T, Schlattl A, Stuetz AM, Benes V, Korbel JO (2012). DELLY: structural variant discovery by integrated paired-end and split-read analysis. Bioinformatics.

[65] Zeitouni B, Boeva V, Janoueix-Lerosey I, Loeillet S, Legoix-ne P, Nicolas A (2010). SVDetect: a tool to identify genomic structural variations from paired-end and mate-pair sequencing data. Bioinformatics.

[66] Srivastava A, Philip VM, Greenstein I, Rowe LB, Barter M, Lutz C, et al. Discovery of transgene insertion sites by high throughput sequencing of mate pair libraries. BMC Genomics. 2014;15:367. doi:10.1186/1471-2164-15-367. 10.1186/1471-2164-15-367 PMC4035081 24884803

[67] Guttikonda SK, Marri P, Mammadov J, Ye L, Soe K, Richey K (2016). Molecular characterization of transgenic events using next generation sequencing approach. PLoS One.

[68] Li H, Handsaker B, Wysoker A, Fennell T, Ruan J, Homer N (2009). The Sequence Alignment/Map format and SAMtools. Bioinformatics.

[69] Yang L, Wang C, Holst-Jensen A, Morisset D, Lin Y, et al. Characterization of GM events by insert knowledge adapted re-sequencing approaches. Sci Rep. 2013;3:2839. doi:10.1038/srep02839. 10.1038/srep02839 PMC3789143 24088728

[70] Simpson JT, Wong K, Jackman SD, Schein JE, Jones SJM, Birol I (2009). ABySS: A parallel assembler for short read sequence data. Genome Res.

[71] Segerman B, De Medici D, Schulz ME, Fach P, Fenicia L, Fricker M (2011). Bioinformatic tools for using whole genome sequencing as a rapid high resolution diagnostic typing tool when tracing bioterror organisms in the food and feed chain. Int J Food Microbiol.

[72] DuBose AJ, Lichtenstein ST, Narisu N, Bonnycastle LL, Swift AJ, Chines PS, et al. Use of microarray hybrid capture and next-generation sequencing to identify the anatomy of a transgene. Nucleic Acids Res. 2013;41(6):e70. doi:10.1093/nar/gks1463. 10.1093/nar/gks1463 PMC3616733 23314155

[73] Lepage E, Zampini E, Boyle B, Brisson N. Time- and cost-efficient identification of T-DNA insertion sites through targeted genomic sequencing. PLoS One. 2013;8(8):e70912. doi:10.1371/journal.pone.0070912. 10.1371/journal.pone.0070912 PMC3741346 23951038

[74] Zastrow-Hayes GM, Lin H, Sigmund AL, Hoffman JL, Alarcon CM, Hayes KR, et al. Southern-by-sequencing: a robust screening approach for molecular characterization of genetically modified crops. Plant Genome. 2015;8(1):1–15. doi:10.3835/plantgenome2014.08.0037. 10.3835/plantgenome2014.08.0037 33228291

[75] Marcais G, Kingsford C (2011). A fast, lock-free approach for efficient parallel counting of occurrences of k-mers. Bioinformatics.

[76] Li H, Durbin R (2010). Fast and accurate long-read alignment with Burrows-Wheeler transform. Bioinformatics.

[77] Warren RL, Sutton GG, Jones SJM, Holt RA (2007). Assembling millions of short DNA sequences using SSAKE. Bioinformatics.

[78] Kent WJ (2002). BLAT - The BLAST-like alignment tool. Genome Res.

[79] Willems S, Fraiture M-A, Deforce D, De Keersmaecker SCJ, De Loose M, Ruttink T (2016). Statistical framework for detection of genetically modified organisms based on next generation sequencing. Food Chem.

[80] European Commission (2003). Regulation (EC) no 1829/2003 of the European Parliament and of the Council of 22 September 2003 on genetically modified food and feed. Off J Eur Union.

[81] OECD. OECD guidance for the designation of a unique identifier for transgenic plants ENV/JM/MONO(2002)7/REV1(2006). OECD - Environment Directorate, Joint meeting of the Chemicals Committee and the Working Party on Chemicals, Pesticides and Biotechnology. 2006. http://www.oecd.org/science/biotrack/46815728.pdf. Accessed 5 Feb 2016.

[82] Brodmann P, Ilg E, Berthoud H, Herrmann A (2002). Real-time quantitative polymerase chain reaction methods for four genetically modified maize varieties and maize DNA content in food. J AOAC Int.

[83] EURL-GMFF. GMOMETHODS - EU database of reference methods for GMO analysis. European Union Reference Laboratory for GM Food and Feed, European Commission, Joint Research Centre. 2015. http://gmo-crl.jrc.ec.europa.eu/gmomethods/. Accessed 5 Feb 2016.

[84] European Food Safety Authority (2010). Guidance on the environmental risk assessment of genetically modified plants. EFSA J.

[85] European Food Safety Authority (2011). Guidance for risk assessment of food and feed from genetically modified plants. EFSA J.

[86] Rabbi IY, Kulakow PA, Manu-Aduening JA, Dankyi AA, Asibuo JY, Parkes EY (2015). Tracking crop varieties using genotyping-by-sequencing markers: a case study using cassava (*Manihot esculenta* Crantz). BMC Genet.

[87] Barbau-Piednoir E, Stragier P, Roosens N, Mazzara M, Savini C, Van den Eede G (2014). Inter-laboratory testing of GMO detection by combinatory SYBR ® green PCR screening (CoSYPS). Food Anal Methods.

[88] Morisset D, Novak PK, Zupanic D, Gruden K, Lavrac N, Zel J. GMOseek: a user friendly tool for optimized GMO testing. BMC Bioinform. 2014;15:258. doi:10.1186/1471-2105-15-258. 10.1186/1471-2105-15-258 PMC4138379 25084968

[89] Holst-Jensen A, Bertheau Y, Alnutt T, Broll H, de Loose M, Grohmann L, et al. Guidance document from the European Network of GMO Laboratories (ENGL): overview on the detection, interpretation and reporting on the presence of unauthorised genetically modified materials. Report no. EUR 25008 EN - scientific and technical research series. European Commission, Joint Research Centre, Luxembourg. 2011; 58 pp. doi:10.2788/89665.

